# Emotional Dissonance, Mental Health Complaints, and Sickness Absence Among Health- and Social Workers. The Moderating Role of Self-Efficacy

**DOI:** 10.3389/fpsyg.2018.00592

**Published:** 2018-04-24

**Authors:** Anne-Marthe R. Indregard, Stein Knardahl, Morten B. Nielsen

**Affiliations:** National Institute of Occupational Health, Oslo, Norway

**Keywords:** emotional dissonance, emotion regulation, exhaustion, mental distress, self-efficacy, sickness absence, registry data

## Abstract

Health- and social workers are frequently exposed to emotionally demanding work situations that require emotion regulation. Studies have demonstrated a direct relationship between emotion regulation and health complaints and sickness absence. In order to prevent health complaints and to reduce sickness absence among health- and social workers, there is need for greater attention to mechanisms explaining when and how emotionally demanding work situations are related to employee health and sickness absence. The overarching aim of this study was therefore to examine the moderating role of generalized self-efficacy on the association between emotional dissonance, employee health (mental distress and exhaustion), and registry based sickness absence. The sample consisted of 937 health- and social workers. Data on emotional dissonance, generalized self-efficacy, exhaustion, and mental distress was collected through questionnaires, whereas official registry data were used to assess sickness absence. A two-step hierarchical regression analysis showed that emotional dissonance was significantly associated with exhaustion, mental distress, and sickness absence, after adjusting for sex, age, and occupation. Interaction analyses with simple slope tests found that self-efficacy moderated the association between emotional dissonance and both exhaustion and mental distress, but not the association with sickness absence. This study shows that health- and social workers who frequently experience emotional dissonance report higher levels of exhaustion and mental distress, and have a higher risk of medically certified sickness absence. Further, health- and social workers with lower self-efficacy beliefs are apparently more sensitive to the degree of emotional dissonance and experienced higher levels of exhaustion and mental distress.

## Introduction

Health- and social workers are frequently exposed to emotionally demanding work situations when they provide support and assistance to patients and clients. The emotional aspects of working directly with patients and clients, *emotion work* (also known as “emotional labor”), refer to psychological processes necessary to express emotions that are desired by the organization during interactions (Zapf, 2002). In her seminal book, *The Managed Heart*, Hochschild (1983) proposed emotional labor as a work stressor that is potentially detrimental to the psychological and physical well-being of employees. Later, studies have shown that emotion work, and especially experiencing a discrepancy between felt and expressed emotions, *emotional dissonance*, can contribute to strain (Zapf et al., 1999; Grandey, 2003), and increase the risk of adverse psychological outcomes (Zapf et al., 1999; Zapf, 2002). In sociology, the term emotional labor refers to the exchange value of work and emotion work to the use value in private contexts. In psychology, the term labor is often used when sociological and social concepts are involved and not when individual behaviors, such as having to regulate emotions at work, are the concept of interest. Therefore, in line with Zapf (2002) and the field of work psychology, the term emotion work is preferred in the current study.

**FIGURE 1:**
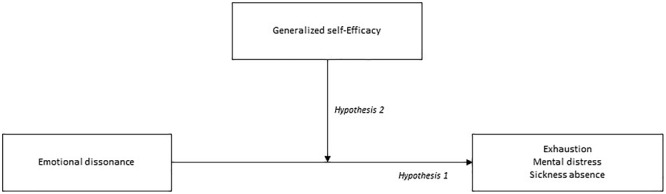
A graphical conceptual overview of the described moderated associations.

Much of the research conducted with health- and social workers have focused on the influence of environmental work factors (Schaufeli and Enzmann, 1998), but it is also important to consider how different individual factors are associated with employee health and well-being (Taris and Schaufeli, 2015). It is unlikely that all employees respond to emotional dissonance in the same manner, and individual differences among the employees may explain variation in the outcomes. Generalized self-efficacy, defined as a broad and stable sense of personal competence to deal effectively with a variety of stressful situations (Jerusalem and Schwarzer, 1992), has been proposed as one important individual disposition that may determine the impact of work stressors on health (Schreurs et al., 2010). High level of self-efficacy has been related to higher self-esteem, better well-being, better physical condition, and better adaptation to and recovery from acute and chronic diseases (Bandura, 1997). In contrast, individuals with low self-efficacy are more likely to suffer from distress and negative emotions, such as anxiety, depression, helplessness, and burnout (Schwarzer and Hallum, 2008). To add to the understanding of how self-efficacy may influence the association between emotion work and health, the overarching aim of this study was to investigate the moderating role of self-efficacy, on the relationships between emotional dissonance, employee health (mental distress and exhaustion), and registry-based sickness absence. A graphical overview of the described moderated associations is included in **Figure 1**. As the aim of this study was to determine the moderating effect of generalized self-efficacy on outcomes of emotional dissonance, it should be noted that we do not propose, or test, the causal associations with exhaustion, mental distress, and sickness absence in the current data.

### Emotional Dissonance, Mental Health, and Sickness Absence

Emotional dissonance is one dimension of emotion work and considered as a stressor present in client-driven work environments (Zapf, 2002). Although the causal relationships between emotional dissonance and employee health needs to be further clarified, it has been suggested that emotional dissonance may lead to negative health outcomes due to costs of regulating emotions in order to display the desired emotion (Zohar et al., 2003; Grandey and Melloy, 2017). Managing emotional expressions as part of one’s job is described as a complex, transient, and dynamic interpersonal process (Grandey and Melloy, 2017) which includes all of the efforts to increase, maintain, or decrease one or more components of an emotion (Gross, 1999). Thus, in line with the health impairment process (Bakker and Demerouti, 2007; Ceschi et al., 2016), regulating emotions to express a desired display may be an effortful process that drain mental resources and thereby enhances strain (Grandey, 2003; Coté, 2005). In an occupational context, strain may be defined as a set of psychological, physiological, and behavioral reactions to work stressors (Coté, 2005). This definition is in line with prior studies that have demonstrated that experiencing emotional dissonance increases the risk of feeling exhausted (Zapf, 2002; Hülsheger and Schewe, 2011), being psychologically distressed (Diestel and Schmidt, 2011), and being absent from work (Nguyen et al., 2013; Indregard et al., 2016).

Emotional exhaustion, the key component of burnout, was originally related to client-work situations (Maslach and Jackson, 1981), and it has frequently been studied among health- and social workers. Exhaustion describes a sense of feeling psychologically and emotionally “drained” and some early studies indicated that exhaustion might be a result of long-term exposure to excessive job demands and continuous hassles (Lee and Ashforth, 1996; Zohar, 1997). Symptoms of depression and anxiety (mental distress) are not defined as related to a specific context, and are less studied in relation to emotion work compared to exhaustion. However, studies have demonstrated that social and psychological work factors increases the risk of experiencing mental distress (Finne et al., 2014). The present study therefore included both measures of exhaustion and mental distress in addition to medically certified sickness absence, as indicators of impaired employee health. We hypothesized that health- and social workers that frequently experience emotional dissonance at work have a higher risk of feeling exhausted, experience mental distress, and are at higher risk of being absent from work.

Thus, we expect that emotional dissonance is positively related to exhaustion, mental distress, and risk of sickness absence (hypothesis 1: H1).

However, this H1 will not explain when and for whom emotional dissonance can lead to negative health outcomes. By focusing on the moderating role of generalized self-efficacy, the present study will investigate an individual factor in order to provide more knowledge about the direct relationships between emotional dissonance and employee health and sickness absence.

### The Moderating Role of Self-Efficacy

Most health- and social workers will experience situations that call for activation or suppression of emotions that may be in conflict with truly felt emotions. However, this discrepancy between felt and expressed emotions does not necessarily need to result in impaired health and well-being (Heuven and Bakker, 2003; Coté, 2005; Heuven et al., 2006). That is, employees may use several strategies when regulating their emotions, leading to various emotion regulation processes and different outcomes (Grandey and Melloy, 2017). Consequently, these individual differences may affect the stressor-strain relationship between emotional dissonance and employee health and well-being.

With regard to specific individual factors that may be of importance, prior studies have showed that self-efficacy may have a buffering effect by decreasing the negative consequences of performing emotion work (Heuven et al., 2006). According to social cognitive theory (Bandura, 1986), self-efficacy reduces distress and increases motivation when facing difficult, novel or threatening tasks, such as emotionally charged patient interactions. Employees with high levels of self-efficacy are found to be generally better to effectively and successfully use and generate resources in their working environment and to employ different and more effective coping strategies than individuals low in self-efficacy (Consiglio et al., 2013). Whereas self-efficacy is commonly understood as being task- or domain-specific (Bandura, 1992), other researchers (Jerusalem and Schwarzer, 1992) have introduced a more trait-like version of the concept, termed generalized self-efficacy. Generalized self-efficacy refers to a stable belief in the ability to deal efficiently with a wide range of stressors and may be conceived as a personal resource in a stress process. In this respect, generalized self-efficacy can be viewed as a moderator of the relationships between a stressor such as emotional dissonance and employee health.

Based on these theoretical considerations and empirical evidence, we argue that negative health outcomes may result from an interaction between experiencing emotional dissonance and the employee’s level of generalized self-efficacy, i.e., beliefs about their competence to deal with a stressful situation. We therefore hypothesized that generalized self-efficacy buffers the relationships between emotional dissonance and mental health complaints (exhaustion and mental distress) and sickness absence.

Thus, we expect that the positive relationships between emotional dissonance and mental health complaints and sickness absence are moderated by self-efficacy, i.e., that the relationships are weaker for employees with high levels of self-efficacy (H2).

## Materials and Methods

### Study Sample and Design

The current study was a study of Norwegian employees who participated in a comprehensive prospective study: “The new work place: Work, health, and participation in the new work life,” a longitudinal web-based survey carried out by the National Institute of Occupational Health (see Christensen and Knardahl, 2010; Finne et al., 2014; Emberland and Knardahl, 2015). All psychological and social work factors were measured at baseline, and then linked to official registry data on sickness absence for the year following the survey assessment. For a more detailed description of the research project, see study protocol published elsewhere (Nielsen et al., 2016).

Recruitment and data collection took place from November 2004 to December 2014. Organizations were contacted by the National Institute of Occupational Health and offered to participate in the study. After information about the general study aims was given at the organizational level, each employee, excluding those on sick leave, received a letter containing information about the survey, the strict confidentiality guidelines, as well as information about the license for data collection granted by the Norwegian Data Inspectorate. A written consent was obtained before linking survey questionnaire to registry data on sickness absence. A detailed description of the recruitment has been published elsewhere (Christensen and Knardahl, 2010).

Some organizations were contacted by the National Institute of Occupational Health (NIOH) and offered to participate in the study, whereas other organizations contacted NIOH themselves in order to participate in the study. Altogether 15,302 persons responded (response rate: 49.4%). The current study sample consisted of 937 health- and social workers (*registered nurses n* = 331 (35%), *health care assistants, including enrolled nurses n* = 448 (48%), *social workers n* = 69 (7%), *physicians n* = 19 (2%), and *other health care professions, such as physical- and occupational therapists n* = 70 (8%). The sample consisted mostly of women (91.1%), and the mean age was 44.1 years, *SD* = 11.0. About 53.5% had minimum 13 years of education, 84.2% were permanently employed, and the majority did not have management responsibilities (87.1%).

### Measures

Data on emotional dissonance, exhaustion, mental distress, self-efficacy, and control variables were collected thorough the questionnaire survey, whereas we used official registry data to assess sickness absence. The questionnaires were available in both Norwegian and English.

#### Emotional Dissonance

Emotional dissonance was measured by five items (α = 0.89) adapted from the Frankfurt Emotion Work Scales (Zapf et al., 1999), example item: “How often in your job do you have to suppress emotions in order to appear neutral on the outside?”. Responses were provided on a five point scale with the following alternatives 1 = seldom or never, 2 = once per week, 3 = once per day, 4 = several times per day, and 5 = several times an hour. Evidence for criterion-related validation of the scale has been showed by Zapf et al. (1999). To validate the Norwegian translation of the scale, an independent back-translation to German was performed. The back translation showed good conceptual equivalence with the original version (Indregard et al., 2016).

#### Exhaustion

A sub-dimension from the Copenhagen Burnout Inventory (CBI) (Kristensen et al., 2005) was used to measure exhaustion, example items: “How often do you feel tired?” and “How often do you feel worn out?”. The dimension, personal burnout, consists of six questions measuring exhaustion. Cronbach’s α was 0.84. Personal burnout is regarded as a state of prolonged physical and psychological exhaustion (Winwood and Winefield, 2004). The measurement does not attempt to distinguish between physical and psychological exhaustion and the experience of exhaustion is not a phenomenon restricted to human service professions (Kristensen et al., 2005). The answer was scored on a scale from 1 to 5, where 1 = very seldom or never, and 5 = nearly every day.

#### Mental Distress

Degree of mental distress (symptoms of anxiety and depression) during the last week was measured by a Norwegian translation of the Hopkins Symptoms Checklist-10 (HSCL-10), example items: “feeling tense or keyed up,” “feeling of worthlessness,” and feeling fearful.” HSCL has demonstrated adequate psychometric properties (Derogatis et al., 1974), and is a frequently used self-report instrument to assess mental distress in population surveys. Responses are given on a scale from 1 to 4: 1 = not at all, 2 = a little, 3 = quite a bit, and 4 = extremely. The study used mental distress as a continuous scale in the analyses. Cronbach’s α for the scale was 0.87.

#### Self-Efficacy

Degree of self-efficacy was measured by three questions (α = 82) adopted from The Generalized Self-Efficacy Scale (Schwarzer and Jerusalem, 1995). The scale was created to assess a general sense of perceived self-efficacy with the aim in mind to predict coping with daily hassles as well as adaptation after experiencing all kinds of stressful life events. The three questions used in the present study was: (1) I can solve most problems if I invest the necessary effort; (2) When I am confronted with a problem, I can usually find several solutions, and (3) I can usually handle whatever comes my way. Responses were given on a five point scale from 1 = strongly disagree to 5 = strongly agree.

#### Sickness Absence

Registry data on medically certified sickness absence was retrieved through the Norwegian Labor and Welfare Administration (NAV). The registry provides complete registrations of all medically certified sickness absence from the first day absent, including the length and medical diagnosis. The registry should be accurate since correct registration is required for the transfer of payments by the social insurance scheme. We aggregated data on sickness absence over a 12-month follow-up post survey, which is consistent with previous research (Diestel and Schmidt, 2010; Nguyen et al., 2013). Registry information of sickness absence was linked to the survey data by the unique 11-digit national individual identity number. The time period the employees were eligible for sickness absence was considered the same for all respondents within each company, starting from the day the electronic forms were closed. The registry was checked for inconsistencies. Overlapping or duplicate spells of sickness absence were merged.

### Statistical Analyses

Statistical analyses were performed using IBM SPSS Statistics 25.0 and PROCESS macro 3.0 (Hayes, 2017). For all questionnaire inventories, summary scales were calculated based on a mean-score of their respective items. Medically certified sickness absence was used as a dichotomous variable (0 = no medically certified sickness absence; 1 = one or more days of medically certified sickness absence). The study implements a cross-sectional design when analyses the exhaustion and mental distress as outcomes and a prospective design when sickness absence is the outcome. To test our hypotheses, we first ran traditional two-step hierarchical regression analyses using IBM SPSS. In the first step, control variables (i.e., age, sex, and occupation), emotional dissonance, and self-efficacy were entered to investigate the main effects. In the second step, the multiplicative interaction term (emotional dissonance × self-efficacy) was entered to directly test the moderating effect of self-efficacy. The moderator hypothesis is supported if the interaction term is significant. To further investigate the nature of the moderation, we performed simple slope tests using PROCESS macro 3.0. We obtained regions of significance with the Johnsen-Neyman technique that yields statistical significance transitions points within the observed range of the moderator. Finally, we plotted conditional effects (simple slopes) for low (sample mean – 1 SD) and high (sample mean + 1 SD) levels of the moderator (self-efficacy). The scores on each predictor variables were mean centered to aid the interpretability of the results.

## Results

### Descriptive Statistics

Overall, 86% of the employees reported that they have personal contacts very often or always during a working day, and in average they spend 4–6 h in direct contact with patients or clients every day. As many as 67% reports that they rarely can decide themselves when to have contact with patients or clients. Altogether 49.5% had at least one day with medically certified sickness absence within the year following the survey measurement.

**Table 1** presents descriptive statistics and intercorrelations for the study variables. Emotional dissonance was significantly positive correlated with exhaustion (*r* = 0.23, *p* < 0.01), mental distress (*r* = 0.19, *p* < 0.01), sickness absence (*r* = 0.08, *p* < 0.05), and significantly negative correlated with self-efficacy (*r* = -0.11, *p* < 0.01). Exhaustion was significantly positive correlated with mental distress (*r* = 0.73, *p* < 0.01) and sickness absence (*r* = 0.12, *p* < 0.01), and significantly negative correlated with self-efficacy (*r* = -0.13, *p* < 0.01). Mental distress was significantly positive correlated with sickness absence (*r* = 0.12, *p* < 0.01), and negative correlated with self-efficacy (*r* = -0.19, *p* < 0.01).

**Table 1:** Means (M), standard deviations (SD), and intercorrelations for study variables.

Variables	Descriptive		Correlations				
	*M*	*SD*	1	2	3	4	5
1. Emotional dissonance	2.80	0.91	–				
2. Exhaustion	1.93	0.73	0.23**	–			
3. Mental distress	1.42	0.41	0.19**	0.73**	–		
4. Self-efficacy	3.83	0.46	–0.11**	–0.13**	–0.19**	–	
5. Sickness absence			0.08*	0.12**	0.12**	–0.02	–

*Sickness absence is scored as 0 (having no sickness absence) and 1 (having one or more days with sickness absence). ^∗^*p* < 0.05, ^∗∗^*p* < 0.01.*

To test common method variance (CMV), we performed a Harman’s (1976) single factor test. Results showed that the total variance for one single factor was 28.7%, indicating that CMV is not likely to influence our results.

### The Impact of Emotional Dissonance and Self-Efficacy on Exhaustion, Mental Distress, and Sickness Absence

The main effects of emotional dissonance on exhaustion, mental distress, and sickness absence were entered into the equation in Step 1 of the hierarchical regression analysis. **Table 2** shows a significant main effect of emotional dissonance on exhaustion (*b* = 0.18, 95% CI = 0.12, 0.23; *p* < 0.01) and on mental distress (*b* = 0.08, 95% CI = 0.05, 0.11; *p* < 0.01), and **Table 3** presents a significant main effect of emotional dissonance on sickness absence (OR = 1.16, 95% CI = 1.00, 1.36; *p* = 0.02). In Step 2, the interaction of emotional dissonance and self-efficacy was included in the regression analysis. The interaction term significantly predicted both exhaustion (*b* = -0.11, 95% CI = -0.20, -0.01; *p* = 0.02) and mental distress (*b* = -0.06, 95% CI = -0.11, -0.01; *p* = 0.03). In addition, as displayed in **Table 2**, including the interaction of emotional dissonance and self-efficacy significantly explained additional variance in predicting both exhaustion (Δ*R*^2^= 3%) and mental distress (Δ*R*^2^= 5%). The interaction between emotional dissonance and self-efficacy proved no significant effect in predicting sickness absence (**Table 3**).

**Table 2:** Estimates of the main and interaction effects of emotional dissonance and self-efficacy on exhaustion and mental distress.

Variables	Exhaustion		Mental distress	
	*b*	95% CI	*b*	95% CI
*Step 1*				
Emotional dissonance (ED)	–0.18^∗∗^	–0.12, –0.23	0.08^∗∗^	0.05, 0.11
Self-efficacy (SA)	–0.16^∗∗^	–0.25, –0.07	–0.14^∗∗^	–0.18, –0.08
	*R*^2^	*F*	*R*^2^	*F*
	0.067	12.19 (5,873)^∗∗^	0.076	14.65 (5,889)^∗∗^
*Step 2*				
Emotional dissonance	0.59^∗^	–0.21, 0.98	0.32^∗∗^	0.10, 0.53
Self-efficacy	0.14	–0.14, 0.43	0.03	–0.12, 0.19
ED × SA	–0.11^∗^	–0.20, –0.01	–0.06^∗^	–0.11, –0.01
	*R*^2^	*F*	*R*^2^	*F*
	0.097	28.78 (1,872)^∗∗^	0.127	51.80 (1,888)^∗∗^

*Sickness absence is scored as 0 (having no sickness absence) and 1 (having one or more days with sickness absence). All analyses are adjusted for sex, age, and occupation; *n* = 889; ^∗^*p* < 0.05; ^∗∗^*p* < 0.01.*

**Table 3:** Estimates of the main and interaction effect of emotional dissonance and self-efficacy on medically certified sickness absence.

Variables	Sickness absence	
	*OR*	95% CI
*Step 1*		
Emotional dissonance (ED)	1.16^∗^	1.00, 1.35
Self-efficacy (SA)	0.97	0.76, 1.25
*Step 2*		
Emotional dissonance	0.84	0.30, 2.38
Self-efficacy	0.77	0.35, 1.68
ED × SA	1.09	0.84, 1.41

*Adjusted for sex, age, and occupation. ^∗^*p* < 0.05.*

**FIGURE 2:**
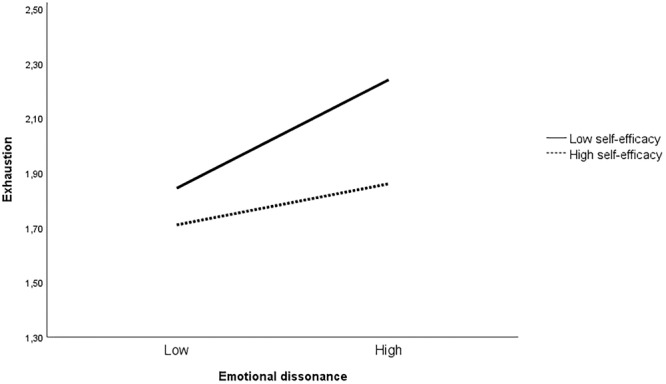
The moderating effect of self-efficacy on the relationship between emotional dissonance and exhaustion (*n* = 879).

**FIGURE 3:**
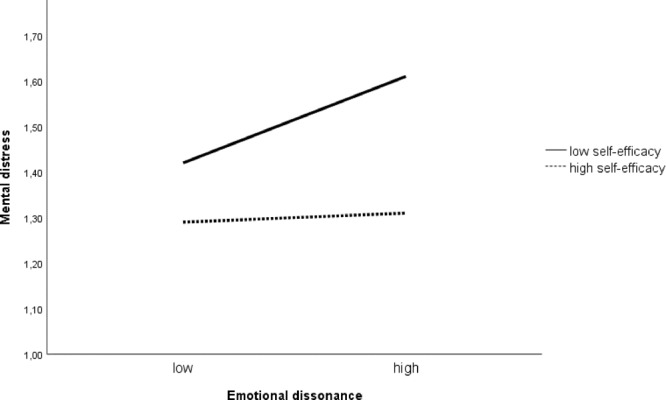
The moderating effect of self-efficacy on the relationship between emotional dissonance and mental distress (*n* = 879).

As displayed in **Figures 2, 3**, the significant interaction of emotional dissonance and self-efficacy was decomposed by computing simple slopes of emotional dissonance on high and low levels of self-efficacy (1 SD above and 1 SD below the mean). Results revealed that the relationship between emotional dissonance and exhaustion, and between emotional dissonance and mental distress, was significantly stronger for employees with low self-efficacy. Thus, H2 was partly supported: self-efficacy moderated the impact of emotional dissonance on exhaustion and mental distress, but not the association between emotional dissonance and sickness absence. The Johnson-Neyman test revealed that the effect of self-efficacy on the relationship between emotional dissonance and exhaustion was significant when the value was 0.75 and below. For the relationship between emotional dissonance and mental distress, the threshold value for significance was at 0.53 and below.

## Discussion

The current study contributes to the literature on emotion work by elucidating effects of generalized self-efficacy that may influence relationships between emotional dissonance, employee health, and sickness absence. Health- and social workers who frequently experience emotional dissonance at work reported higher levels of both exhaustion and mental distress. While prior studies have demonstrated that experiencing emotional dissonance can lead to exhaustion (Zapf, 2002; Hülsheger and Schewe, 2011), the present study showed that emotional dissonance was also associated with mental distress. Exhaustion and mental distress (i.e., symptoms of anxiety and depressions) are related (Bianchi et al., 2015; Schonfeld and Bianchi, 2016) and we found a rather high correlation of 0.73 between exhaustion and mental distress. Although the confidence intervals from the regression estimate indicate an overlap in magnitude, emotional dissonance was more strongly associated with exhaustion than with mental distress in the present study. An explanation for this finding may be that the threshold for reporting symptoms of exhaustion, such as tiredness and being worn out, may be lower than that for symptoms of depression and anxiety, as these latter symptoms may be perceived as graver by the respondents.

Experiencing emotional dissonance was found to increase the risk of medically certified sickness absence. This finding is supported in prior studies (Diestel and Schmidt, 2011; Nguyen et al., 2013) and consistent with the theoretical association between emotion regulation and absenteeism proposed by Grandey (2000). Regulating emotions may strain the employee and absenteeism may be a coping strategy to prevent being subjected to aversive situations at work (Edwards, 1991; Grandey, 2000).

While emotional dissonance was significantly associated with exhaustion, mental distress, and sickness absence, the results from the interaction analyses nuanced our findings by establishing individual differences in the associations. Specifically, the interaction analyses showed that self-efficacy significantly moderated the association between emotional dissonance and exhaustion and mental distress, but not the association between emotional dissonance and sickness absence: higher levels of emotional dissonance were associated with high levels of exhaustion and distress among those with low self-efficacy. Those with high levels of self-efficacy reported low levels of both exhaustion and distress irrespectively of levels of emotional dissonance. This is in line with the study by Pugh et al. (2011) showing that employees feeling confident about their ability to manage their emotional display are less negatively affected when experiencing emotional dissonance. Prior studies have demonstrated that the resource-depleting effects of emotion regulation processes are not the same for everyone (Nguyen et al., 2013) and self-efficacy is considered an important personal resource enabling employees to meet emotional demands (Hobfoll and Shirom, 2001). In the context of performing emotion work, health- and social workers with high self-efficacy are likely to believe in their ability to regulate emotions in interpersonal relations and having to display a variety of emotions is less likely to be experienced as a drain of energy (Wilk and Moynihan, 2005). In a prior study by Consiglio et al. (2013), they found that employees with higher levels of self-efficacy are more able to generate available resources and to cope with demands in their working environment.

### Limitations and Further Studies

The cross-sectional study design limits any conclusions about cause and effect relationships. That is, with only one survey time point, we were only able to determine whether emotional dissonance was associated with exhaustion and mental distress and the findings should therefore not be used to draw conclusions about the causal order of the variables. However, as sickness absence was assessed with time-specific registry data, respondent’s exposure to emotional dissonance took place before the sickness absence and this may thereby imply a causal association.

Strengths of this study are the use of psychometrically sound measurement instruments to measure the emotional dissonance, exhaustion, mental distress, and self-efficacy, and the use of official registry data to assess sickness absence. The response rate was 49.4% and above the average level established for organizational surveys (Baruch and Holtom, 2008). Participating organizations were recruited through convenience sampling methods, which limit the external validity of the findings (Mazzocchi, 2008). However, it should be noted that all employees in the participating organizations were invited to participate in the survey. The sample can therefore be considered as a probability sample at the individual level (Ilies et al., 2011).

Because the questionnaire instruments were self-report measures, the study could be influenced by bias such as response-set tendencies and social desirability. In addition, the use of self-report measures implies a risk of CMV, i.e., “variance that is attributable to the measurement method rather than to the constructs the measures represent” (Podsakoff et al., 2003, p. 879). When sickness absence is analyzed as outcome, the present study obtains measures of the predictor- and criterion variables from different sources and precludes the risk of observing spurious associations that could be attributed to CMV (Podsakoff et al., 2003). Furthermore, The Frankfurt Emotion Work Scale, used to assess emotional dissonance, does not address issues that are inherently positive or negative. The respondents were asked how often a situation occurs instead of degrees of satisfaction or agreement and the measurement should therefore be insensitive to respondents’ emotions or personality dispositions. Communicating respondent anonymity should also reduce CMV (Podsakoff et al., 2003) and this was prioritized when information about the study were given. In addition, the result from the Harman’s single factor test indicated that CMV is not likely to influence our results.

## Conclusion and Implications

Taken together, the findings of this study show that health- and social workers who frequently experience a discrepancy between felt and expressed emotions also report higher levels of exhaustion and mental distress and have a higher risk of medically certified sickness absence. Further, the results show that health- and social workers with lower self-efficacy beliefs are apparently more sensitive to the degree of emotional dissonance and experienced higher levels of exhaustion and mental distress. In order for organizations to protect employee health and well-being and to prevent sickness absence, an important implication of the study findings is that employers should be aware of the potential detrimental impact of emotional dissonance. As workers with high levels of self-efficacy may be more resilient toward this kind of exposure, employers should be especially aware of the working conditions of employees with low self-efficacy. In doing so, it is important to emphasize that high self-efficacy may be a double-edged sword. On the one hand, self-efficacy is a trait-like, but malleable, individual disposition that can be trained and developed (Luthans et al., 2007) and employers could therefore benefit from interventions that can strengthen the self-efficacy of health- and social workers. As showed in a recent study by Costantini et al. (2017), positive workplace interventions could improve an employee’s psychological capital. On the other hand, previous research findings indicate that persistent exposure to a given work stressor may be detrimental even for those with a highly robust personality (Nielsen et al., 2008; Ilies et al., 2011; Britton et al., 2012; Reknes et al., 2016). Organizations should therefore not only rely on the individual dispositions of their employees alone, but also provide organizational resources that may help their workers deal with the potential impact of emotional dissonance, such as a supportive organizational climate (Kinman et al., 2011; Ortiz-Bonnín et al., 2016).

## Ethics Statement

This project has been approved by the Regional Committees for Medical and Health Research Ethics (REC) in Norway (REC South East), had permission from The Norwegian Data Protection Authority, and was conducted in accordance with the World Medical Association Declaration of Helsinki. All study participants provided their informed consent.

## Author Contributions

A-MI contributed to the design of the study, performed the analysis and interpretation of the data, and wrote the first draft of the manuscript. SK contributed to the design of the study, the data collection, and critically revised the manuscript. MN contributed to the design of the study, contributed to the statistical analysis, and critically revised the manuscript.

## Conflict of Interest Statement

The authors declare that the research was conducted in the absence of any commercial or financial relationships that could be construed as a potential conflict of interest.
